# An Evaluation of Maximum Determination Methods for Center Line Slope Analysis

**DOI:** 10.1021/acs.jpcb.2c07565

**Published:** 2023-05-09

**Authors:** Mason L. Valentine, Garret D. Wiesehan, Wei Xiong

**Affiliations:** Department of Chemistry and Biochemistry, University of California San Diego, San Diego, California 92093, United States

## Abstract

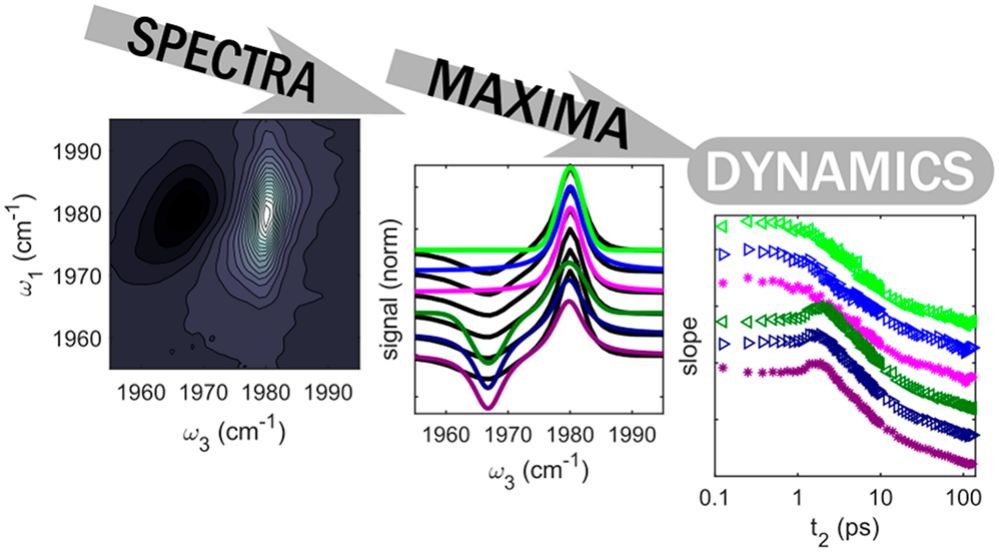

Ultrafast molecular dynamics are frequently extracted from two-dimensional (2D) spectra via the center line slope (CLS) method. The CLS method depends on the accurate determination of frequencies where the 2D signal is at a maximum, and multiple approaches exist for the determination of that maximum. Various versions of peak fitting for CLS analyses have been utilized; however, the impact of peak fitting on the accuracy and precision of the CLS method has not been reported in detail. Here, we evaluate several versions of CLS analyses using both simulated and experimental 2D spectra. The CLS method was found to be significantly more robust when fits were used to extract the maxima, particularly fitting methods that utilize pairs of opposite-sign peaks. However, we also observed that pairs of opposite-signed peaks required more assumptions than single peaks, which are important to check when interpreting experimental spectra using peak pairs.

## Introduction

Coherent two-dimensional (2D) spectroscopy has evolved into an indispensable tool for interrogating dynamics on molecular length and time scales, with a variety of techniques spanning the ultraviolet to terahertz regions of the electromagnetic spectrum,^1−6^ including various interfacial and surface sensitive versions.^4,7−9^ These techniques are utilized to probe the dynamics of systems as diverse as metal organic frameworks,^10^ liquids,^11−13^ polymers,^14^ minerals,^15^ polaritons,^16,17^ excitons,^2,18^ and proteins.^19,20^ One important application of 2D spectra is measuring the frequency–frequency correlation function (FFCF), which describes the time-dependent fluctuations and oscillations of a spectral frequency around a mean value.^21^ The FFCF reports on molecular motions and interactions, e.g., hydrogen bond, electrostatic, hydrophobic and hydrophilic interactions, on the length scale of Angstroms to nanometers, and at the time scale of femtoseconds to hundreds of picoseconds. Further, when combined with computer simulations, the FFCF can yield an even more detailed molecular-level picture.^22^

The FFCF is usually obtained by so-called spectral diffusion measurements, in which a set of 2D spectra are scanned as a function of waiting time (*t*_2_). Multiple techniques exist for the extraction of FFCF parameters from *t*_2_-dependent 2D spectra, each with relative advantages and disadvantages.^21,23,24^ The most widely used method to date is the center line slope (CLS) method,^24,25^ which involves fitting a line to the ridge formed by the maxima of a 2D spectrum, calculating the slope of that line, and observing *t*_2_-dependent changes to the slope. The advantages of the CLS method include ease of implementation and insensitivity to several experimental complications in 2D spectroscopy, including apodization, anharmonicity, and broad background signals.^25,26^ However, the CLS method can begin to lose accuracy with even small amounts of experimental noise.^27^ This represents a significant obstacle, as a full spectral diffusion experiment scans to long *t*_2_ delays, at which it could take hours or days to get sufficient 2D spectra with acceptable signal-to-noise ratios (SNR),^28,29^ limiting the type of sample that is viable for CLS measurements. In cases where noise is present, Kubo Lineshape models can be used to extract the FFCF with an extremely high degree of accuracy and reproducibility (even when compared to the CLS method in the absence of noise).^27^ Kubo Lineshape fitting shows particular advantages for measuring the amplitudes of FFCF components and the dephasing times. However, Kubo Lineshapes are idealized and may not represent complex lineshapes, such as those with non-Condon effects,^30^ highly overlapped peaks,^31,32^ or non-Gaussian fluctuations.^33^ In such cases, the CLS method has been used to measure correlation times (many citations needed).

The most common implementation of the CLS method involves finding the maximum signal at each pump frequency near the center of a peak.^26^ The maximum can be taken directly from the data as the frequency with the maximum measured signal; however, some groups have reported fitting each pump slice to a simple function, such as Gaussian or pseudoVoigt peaks,^34−36^ and then using the fit’s maximum instead of taking the maximum directly from the raw data. It is likely that a fitting approach to CLS is less sensitive to noise than taking the maximum directly. Additionally, the center of mass may be used to determine the maximum under the assumption of a relatively symmetric peak. However, these comparisons are missing from the available literature, and it is not clear which set of functions yields the most accurate FFCF results.

Here we compare various versions of the CLS method and evaluate the usefulness of several functions for the pump slice fits. We first measure the accuracy of each method using simulated 2D IR experiments where the correct FFCF time constants are known exactly and can be compared to CLS results. The effects of simulated noise are examined specifically. Finally, we apply these methods to an experimental model system. We found that within pump slice fitting, any CLS method based on peak pair fitting was much less sensitive to noise than methods using a single peak, while the choice of peak function did not have a significant impact on the precision of the CLS method.

## Materials and Methods

### Kubo Models of 2D IR Spectra

2D IR spectra are generated through the interaction of three ultrashort laser pulses with an oscillator. We modeled theoretical 2D IR spectra using the Kubo model, which has been utilized extensively in 2D spectroscopy to relate FFCF parameters to spectral lineshapes. The FFCF for an oscillator in 2D IR is typically modeled as the sum of exponential functions.^37^ We utilize a biexponential FFCF with a fast modulation component.
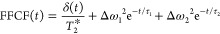
1where *t* is time, δ(*t*) is a delta function and *T*_2_^*^ is the pure dephasing time, which together account for fluctuations too fast to measure. Δω_1_ and τ_1_ are the magnitude and correlation lifetime, respectively, for fluctuations at one time scale, which we refer to as the fast time scale, while Δω_2_ and τ_2_ are the magnitude and correlation lifetime, respectively, for fluctuations at a longer time scale, which we refer to as the slow time scale. The generalized Kubo Lineshape function, *g*(*t*), for this FFCF is written as
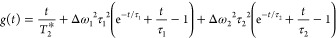
2

The Kubo Lineshape function was used in the response functions that modeled the coherent 2D spectra. Spectra were simulated using the sum of six third-order response functions representing all possible pathways for generating the 2D IR signal in the pump probe phase matching geometry. Response functions accounted for signal from both the transition between ground and first excited states ((01) signal), and the transition between the first and second excited states ((12) signal). The response functions are listed below.
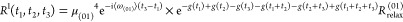
3
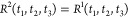
4
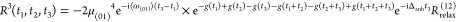
5
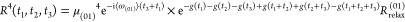
6
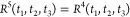
7
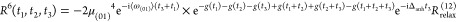
8where i is the square root of −1, μ_(01)_ is the transition dipole moment of the (01) transition, ⟨ω_(01)_⟩ is the mean frequency of the (01) transition, and *t*_1_, *t*_2_, and *t*_3_ are the time delays between the first pump pulse, second pump pulse, probe pulse, and signal, respectively. Δ_anh_ is the anharmonicity of the oscillator, and *R*_relax_^(01)^ and *R*_relax_^(12)^ are the contributions to the response functions from population relaxation for the (01) and (12) signals, respectively. The population relaxation contributions are written below
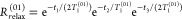
9
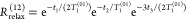
10where *T*_1_^(01)^ is the vibrational lifetime for the first excited state. It should be noted that relaxation in *t*_3_ is different for the (01) and (12) signal. This is because the vibrational lifetime of the second excited state is twice as short as the vibrational lifetime of the first excited state, and also because relaxation of the first and second excited state both contribute to relaxation for the (12) signal.^37^

Response functions were calculated in the time domain, and the real part of the Fourier Transform of the response functions along *t*_1_ and *t*_3_ was used for all analyses. Spectra were calculated with a time domain going out to 120 ps in *t*_1_, *t*_2_, and *t*_3_ with steps of 0.05 ps. This led to a spectral resolution slightly below 0.25 cm^–1^ in both ω_1_ and ω_3_. Spectra were then interpolated to a lower resolution of 0.5 cm^–1^ in ω_1_ and ω_3_.

### Modeling Noise

The largest source of noise for most 2D IR instruments is shot-to-shot fluctuations in laser intensity, although there are some exceptions.^29,38^ Because the acquisition of 2D IR spectra, especially with phase cycling, relies on the subtraction and addition of signals, the shot-to-shot fluctuations of the laser lead to a “baseline wobble” across *t*_1_ and ω_1_. To simulate laser fluctuations, we added a single random number, either positive or negative, to all data points in each pump slice of the final 2D spectrum. Based on the observation that laser fluctuations follow a Gaussian distribution,^38^ we used the randn function in MATLAB to generate the random numbers for simulating the baseline wobble.

Detector noise also contributes to noise in 2D IR spectra, although the amplitude of the detector noise is generally smaller than the amplitude of noise due to laser fluctuations. Since this noise is unlikely to be correlated across ω_1_ or ω_3_, and photon shot noise, which results from the discreteness of photons, is generally negligible for mercury cadmium telluride (MCT) arrays,^38^ detector noise was simulated by adding a different random number from a Gaussian distribution to each data point of the final 2D IR spectrum. The ratio between detector noise and laser noise varies significantly from instrument to instrument due to differing schemes that use reference detectors to suppress laser noise. With referencing, the laser noise could still be about 10 times higher than the detector noise^39^ although recent optimized referencing schemes can bring the laser noise below the detector’s noise floor.^38^ To simulate an intermediate case where there is some referencing, but it is not optimized, we set the laser noise in our simulated data to be a factor of 3 larger than the detector noise.

### Automated CLS Analysis

To test CLS analyses on a large set of simulated data, we automated a standardized version of the CLS method. To sample the fast and slow components of the FFCF, we simulated spectra with 30 logarithmically spaced *t*_2_ values from 0.1 to 200 ps. Pump-slice amplitudes (PSAs)^40^ were used to determine which pump slices to include in the CLS analysis for each spectrum. Only pump slices with a PSA greater than 50% of the spectrum’s maximum PSA were used to extract the center line. The fit to the center line was performed using least-squares fitting with a slope, relative to the pump axis, constrained to be between 0 and 1. The biexponential fit to the CLS decay used a least-squares regression with constraints, and the signs of the exponential terms were constrained to be positive. The fast correlation lifetime was constrained to be between 0.1 and 10 ps, while the slow correlation lifetime was constrained to be between 10 and 100 ps. For experimental 2D IR spectra, the same procedure was used, but with a larger number of *t*_2_ steps and without any constraints on correlation lifetimes during the fits to the CLS decay.

Pump slice fitting used the Levenberg–Marquardt algorithm with a termination tolerance of 10^–10^ for both coefficient and model values. The following function was used for the Gaussian peaks.
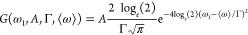
11where *A* is the area under the peak, Γ is the full width at half-maximum (FWHM), and ⟨ω⟩ is the center frequency. The Lorentzian functions were also written in terms of *A*, Γ, and ⟨ω⟩.
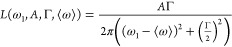
12

The pseudoVoigt functions were written as the weighted sums of the above Lorentzian and Gaussian functions
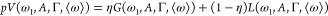
13where η is a weighting factor with a value between 0 and 1.

Estimates of *A*, Γ, and ⟨ω⟩ were used as starting points for the fits. *A* was estimated to be half of the integrated area under the absolute value of each pump slice. Γ was estimated by counting the number of data points in the slice with more than 50% of the maximum signal, and ⟨ω⟩ was estimated using the frequency where the signal was highest. For peak pair fits, the pump slice was modeled as the sum of two copies of the peak. The copies were identical, except that one, the (12) peak, was shifted to lower frequencies by Δ_anh_ and multiplied by −1.

### Experimental 2D Spectra

Saturated W(CO)_6_ in 1-octanol was prepared by placing a 1 mL aliquot of 1-octanol over several milligrams of solid W(CO)_6_ in a glass vial and allowing the solid to dissolve in the dark overnight. A 50 μL aliquot of the solution was held between two 1 in. CaF_2_ windows with a 25 μm PTFE spacer. Prior to measuring 2D spectra we checked to ensure that the optical density was in the appropriate range of 0.3–0.5.

Spectra were collected using an instrument that has been described in detail previously.^16^ Briefly, a titanium-sapphire (Coherent) laser was used to generate pulses centered at 780 nm, which were then converted to the mid-IR region using a commercial OPA (Light Conversion) and a home-built DFG utilizing an AGS crystal. The mid-IR pulses were converted into separate pump and probe pulses using a beam splitter, and the pump was converted to a pulse pair with variable *t*_1_ delays using a pulse shaper with an acousto-optic modulator (Phasetech). A delay stage was used to control *t*_2_. The pump and probe pulses were spatially and temporally overlapped at the sample using a pump–probe geometry and a pair of lenses. The signal and probe pulse were then collimated and a spectrometer was used to disperse the signal and probe onto a 128-by-128 pixel Focal Plane Array (MCT) detector.

Data were collected with perpendicular polarizations for the pump and probe pulses, and *t*_1_ was scanned from 0 to 8.00 ps in 0.032 ps steps. To efficiently capture dynamics on multiple time scales, we utilized nonuniform sampling along *t*_2_ with shorter intervals near *t*_0_. Specifically, we used 0.125 ps *t*_2_ steps from 0.125 to 4.00 ps, 0.25 ps *t*_2_ steps from 4.00 to 10.00 ps, 2.00 ps steps from 10.00 to 30.00 ps, and 5.00 ps steps from 30.00 to 130.00 ps.

## Results and Discussion

### Noise-Free Test of CLS Analyses

Prior to testing slice fitting techniques on a set of noisy spectra, we examined their viability on spectra with no noise added. Simulated spectra were generated from random parameters from the parameter space described in Table 1. Previously, opposite-sign pairs of Gaussian^34,36^ and pseudoVoigt^35^ peaks have been fit to the (01) and (12) signal, and utilized for CLS analysis. As noted by Schneider et al.,^35^ the anharmonicity, which determines the spacing between the (01) and (12) signal, cannot be accurately determined from a single pump slice but can be determined through global fitting of a 2D spectrum to a Kubo model. In our simulations of pump slice fitting, we used the anharmonicity as an input parameter for the peak pair fitting, simulating an experiment where the anharmonicity is known or can be determined from global fitting. The anharmonicity can usually be obtained by fitting 2D spectra to Kubo Lineshape functions but is not always available or constant.^41^ Additional problems with applying peak pairs may arise due to differences between the (01) and (12) lobes of the 2D IR signal. In the simulated spectra used here, these lobes have similar widths, shapes, and intensities due to assumptions implicit in our modeling of the response functions, but these assumptions are not always valid.^37^ Our fitting procedure assumed that the (01) signal and (12) signal had near-identical widths and magnitudes. Without these assumptions, however, fitting 2 different pseudoVoigts to a pump slice could require 6 independent parameters, even with the anharmonicity known (two parameters for peak widths, two parameters for peak amplitudes, two parameters for the relative amplitudes of the Gaussian and Lorentzian components, and one parameter for the center frequency). Thus, pair fits could face quite a few practical challenges.

**Table 1:** Kubo Parameters for Simulated FFCF Experiments

parameter	description	minimum value	maximum value
Δ_anh_	anharmonicity (cm^–1^)	15	60
*T*_1_	vibrational lifetime (ps)	1	100
*T*_2_^*^	pure dephasing time (ps)	0.5	3.0
⟨ω_01_⟩	center frequency (cm^–1^)	2030	2070
Δω_1_	fast fluctuation magnitude (cm^–1^)	2	10
τ_1_	fast fluctuation lifetime (ps)	0.75	3
Δω_2_	slow fluctuation magnitude (cm^–1^)	2	10
τ_2_	slow fluctuation lifetime (ps)	10	40

The large number of parameters that may be necessary for peak pairs led us to also include slice fitting procedures with a smaller parameter space. To accomplish this, we first excluded most of each pump slice from the fit, only applying the fit to data points near the maximum. Any data point with signal less than 50% of the maximum was excluded from the fit. The remaining data were then fit to either a single Gaussian, Lorentzian, or pseudoVoigt function. Single Gaussians have also previously been used for CLS analysis in the literature.^42^

To attempt to extract maxima from pump slices without fitting, we also included the frequencies of maxima (referred to as the direct method) and the peak’s center of mass from the raw data of pump slices. Using the center of mass requires the assumption that the peak is symmetric and unimodal, which is also an implicit assumption when fitting a portion of a pump slice to a single Gaussian, Lorentzian, or pseudoVoigt. The centers of mass were calculated for the absolute values of the total pump slices (full CoM) and for the regions of the pump slices that the single peak fits were applied to (partial CoM).

**Figure 1:**
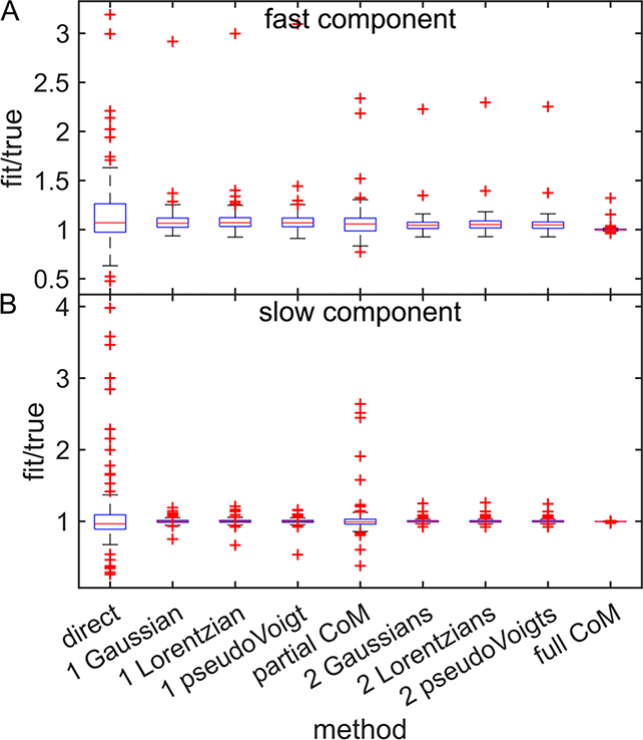
Results from noise-free CLS analysis of test spectra. The median of each distribution is marked with a red line. Box outlines mark the 25th and 75th percentile of each percentile. Whiskers extend to the most extreme data point that is within 1.5 times the interquartile range from the box boundary, and all data points further from the median are marked as red crosses. (A) Box and whisker plot of distributions of estimated fast correlation lifetimes relative to true values. (B) Box and whisker plot of estimated slow correlation lifetimes relative to true values.

The results of the noise-free CLS simulations are shown in Figure 1. The distribution of results is expressed as the ratio between the correlation lifetime measured by the CLS fitting and the true correlation lifetime from the FFCF (fit/true). The mean is shown as a red line, while the box shows the interquartile range, defined by the 25th and 75th percentiles of the distribution. Whiskers extend to the most extreme data point that is within 1.5 times the interquartile range from the box boundary. In a normal distribution, the whisker range would contain 99.3% of the distribution. All points outside of the whiskers are marked as outliers with red crosses.

Differences between the means of these distributions are tested via a one-way analysis of variance (ANOVA), and differences between the spreads of the distributions were tested using the Brown–Forsythe test. In the Brown–Forsythe Test, a distribution of residuals, *z*, is generated according to the following transformation.
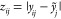
14where *y*_*ij*_ is an individual data point and *ỹ*_*j*_ is the median value from group *j*. Distributions of *z* report on the spreads of the original distributions. A one-way ANOVA test is applied to the *z* distributions, yielding the probability (*p*) that the differences in spread could occur due to random chance. We only reported results as significant if *p* < 0.01.

As shown in Figure 1, all methods yielded a distribution of fit/true values centered near 1; however, the fast lifetime was recovered less accurately than the short lifetime, as the CLS method relies on the short-time approximation and is less reliable for relatively fast processes than for slow processes.^26,27,43^ The full center of mass method was more precise for noise-free simulated CLS than all other methods. The direct method, which just uses the frequency of the pump slice where the signal is highest, was less precise than all other methods. Differences between the direct method and all other methods were determined to be significant (*p* < 0.01) via the Brown–Forsythe test for the fast component. For the slow component, the direct method and partial center of mass were both determined to have significantly more spread in their fit/true distributions than all other methods. The Brown–Forsythe test did not reveal any differences in the spreads of the six different fitting methods. It is worth noting that, even in the absence of noise, none of the methods tested were totally accurate, and all methods except for the full center of mass method had some correlation lifetimes which were off by more than 20%, which points to some of the previously identified issues with the CLS method.^24,27,44^

### Noise Effects on CLS Analyses

To test the CLS methods in the presence of noise, we simulated noisy 2D IR spectra for the same set of 100 systems that were used for the noise-free test and then used the CLS method to estimate the FFCF time constants originally input into the noisy systems. As detailed in the Materials and Methods, we simulated baseline wobble and detector noise in a 3:1 ratio, such that the baseline wobble is significantly larger than detector noise. CLS analysis was performed on each noisy spectrum using all methods that were applied to the noise-free CLS simulations. The estimated correlation lifetimes were then compared to the input parameters as in the noise-free case.

**Figure 2:**
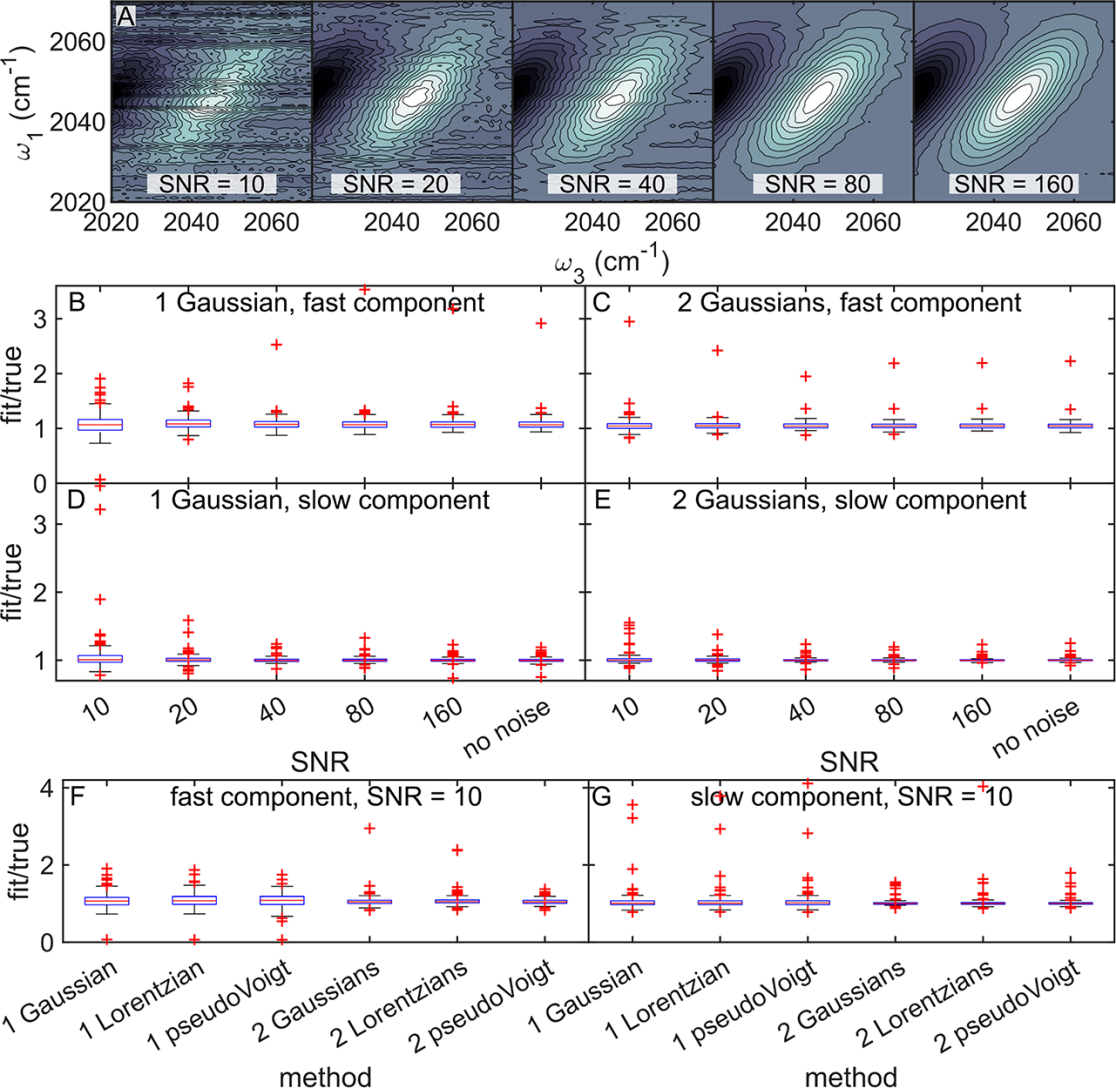
Noise effects on CLS methods. (A) Simulated 2D IR spectrum with SNR from 10 to 160 (B). Box plot of all CLS results for the fast component using single Gaussian fits. (C) Box plot of all CLS results for the fast component using Gaussian pair fits. (D) Box plot of all CLS results for the slow component using single Gaussian fits. (E) Box plot of all CLS results for the slow component using Gaussian pair fits. (F) Box plot of CLS results for the fast component using peak fitting methods at the lowest tested SNR. (G) Box plot of CLS results for the fast component using peak fitting methods at the lowest tested SNR. Noise did not have a significant effect on peak fitting CLS methods for the fast component.

The results of the noise tests are shown in Figure 2 and in Supporting Information Figures S1 and S2. Example spectra at different SNR were shown in Figure 2A. The direct maximum method and the full center of mass were both extremely sensitive to noise, as shown in Supporting Information Figures S1 and S2. This is unsurprising for the direct method, which only uses one pixel from each pump slice. In the case of the full center of mass, sensitivity to noise was largely due to the simulated baseline wobble, as changing the baseline even slightly causes the center of mass of a peak to be drastically different if the baseline is included in the calculation of the center of mass. At all levels of noise, both the full center of mass and the direct maximum performed extremely poorly (see Supporting Information Figures S1 and S2). The partial center of mass, which uses data within the FWHM of each pump slice to calculate the center of mass, performed better than the direct maximum or full center of mass methods, but was sensitive to noise and yielded broader fit/true distributions than fitting.

Peak fitting methods, in contrast, were much less sensitive to noise. As shown in Figure 2B–E, both single and double Gaussian methods were relatively robust toward noise level. Indeed, noise did not have a statistically significant impact on any of the fitting methods for the fast component as demonstrated by the Brown–Forsythe test and ANOVA. For the slow component, there was a significant change in the spread of the fit/true distribution, but the size of the effect was small. Even at the lowest SNR, most of the results from each fitting method remained within ±20% of the true value, as shown in Figure 2F,G. Figure 2 panels F and G shows comparisons between each fitting method for the noisiest simulated data, with SNR = 10. While Gaussians, Lorentzians, and pseudoVoigts all performed similarly, pair fits appeared to yield narrower distributions of fit/true values. We tested the significance of this trend by comparing each single peak method to its pair counterpart using the Brown–Forsythe test. Differences between pair and single peak spreads were only significant for the fast component obtained using pseudoVoigt fitting. There were no significant differences based on model choice at any given SNR. For either single peak or peak pair methods, different fitting functions performed similarly. The only observed trend between fitting methods was that two peaks were better than one.

### Detector Noise Effects on CLS Analyses

**Figure 3:**
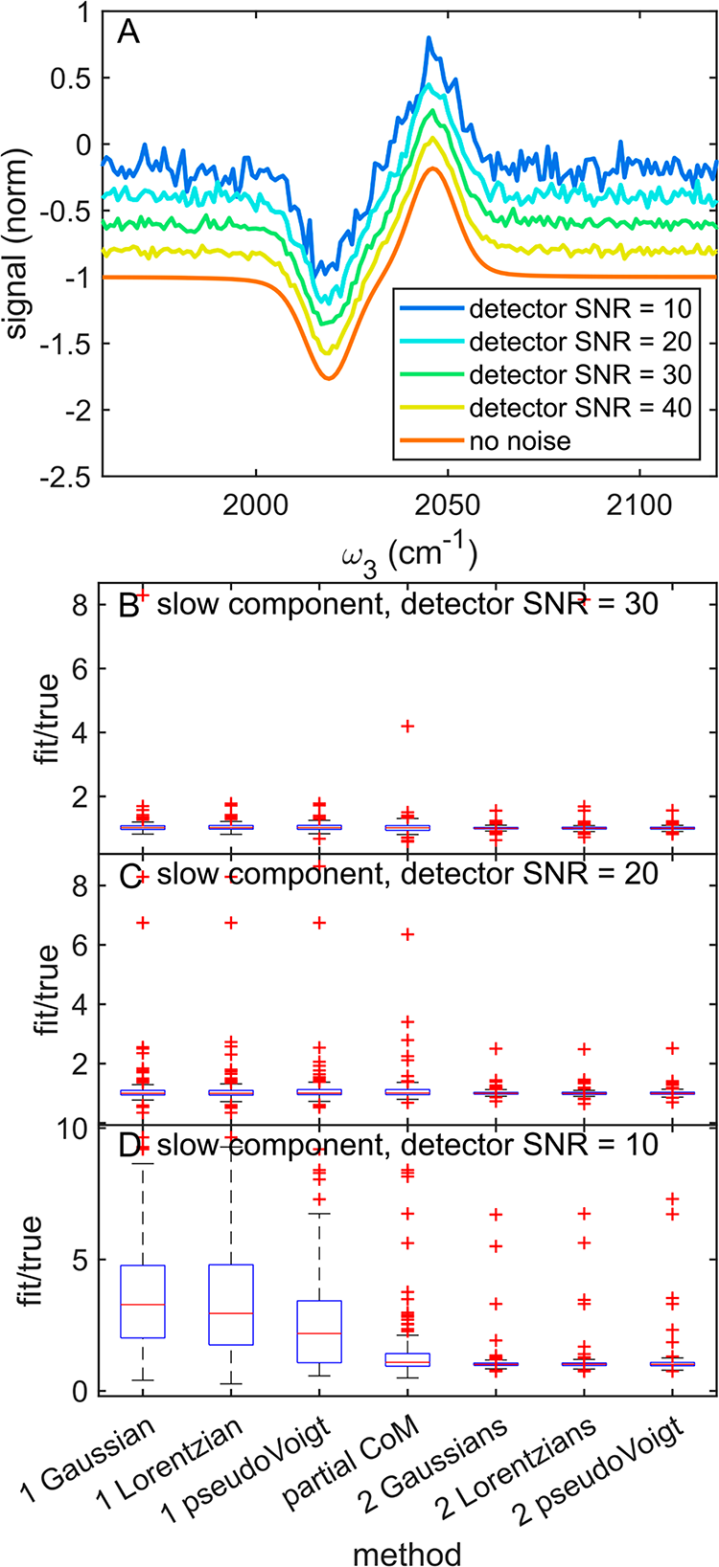
Effects of detector SNR on CLS methods. (A) Representative pump slice with each detector SNR used in the test. (B) Box plot of CLS results for the slow component using fits and the partial center of mass with detector noise set to SNR = 30. (C) Box plot of CLS results for the slow component using fits and the partial center of mass with detector noise set to SNR = 20. (D) Box plot of CLS results for the slow component using fits and the partial center of mass with detector noise set to SNR = 10.

Since some methods and time scales did not show significant sensitivity to noise, we simulated even higher levels of noise for the set of spectra used in previous simulations. In the previous section, simulated baseline wobble noise was three times stronger than simulated detector noise. As a result, the individual pump slices themselves were not noisy. To simulate higher levels of noise and observe the effects of detector noise, we performed an additional test where baseline wobble was held constant at SNR = 10 while detector noise was varied from SNR = 10 to SNR = 40 in increments of 10. Each SNR can be visualized via the representative pump slice shown in Figure 3A, which was taken from a pump frequency where the signal was at a maximum. The full results from this test are shown in Supporting Information S3 and S4. Figure 3 only shows results for the slow component, but the same trends were observed for both the fast and slow components.

When the detector SNR was 30 or 40, we replicated the results from the previous section, as shown in Figure 3B. All fitting methods were significantly more reliable than all nonfitting methods, and peak pairs yielded a narrower fit/true distribution than the corresponding single peaks, as shown in Figure 3B. Again, we note that the difference between peak pairs and single peaks was not always statistically significant at this level of noise. When the detector noise was increased to SNR = 20, as shown in Figure 3C, the single peak fits became less precise, and the differences between peak pairs and single peaks became significant. At this level of detector noise, the partial center of mass, which has been largely ignored at lower levels of noise due to relative inaccuracy, does not yield a significantly broader distribution of fit/true values than the single peak fits.

Figure 3D shows the results from the highest level of detector noise, detector SNR = 10. At this point, the single peak fitting methods became totally unreliable. While the pseudoVoigt single peak appears to have a narrower distribution when compared to the single Gaussian and single Lorentzian, this difference in width was not determined to be significant via the Brown–Forsythe test. The partial center of mass was significantly more precise than the single peak fits for these highly noisy spectra, and the peak pair fitting methods were significantly more precise than the partial center of mass. Most correlation lifetimes obtained using peak pair fits were still within 20% of the true value despite the high level of noise, as shown in the inset of Figure 3D.

Through the simulated tests of noisy CLS experiments, several patterns are clear. First, fits are the most robust methods for determining maxima for CLS analyses at almost every level of noise. Center of mass methods using part of the pump slice may have some utility when noise is extremely high, but these methods are imprecise in the noise-free case and only outperform single peak fits when the detector noise is extremely high. Second, there is no significant difference between peak models. Gaussians, Lorentzians, and pseudoVoigts all perform about the same at every level of noise. Third, peak pairs are less sensitive to noise than single peak fits. This is not surprising, as peak pairs use more detector pixels, and thus a larger amount of data when compared to single peak fits. It should be noted that the biexponential fitting boundaries put a limit on the inaccuracy of the estimated correlation lifetimes, and similar boundaries would not be justified in an experimental setting where the correlation lifetimes are unknown.

### Application to Experimental Spectra

**Figure 4:**
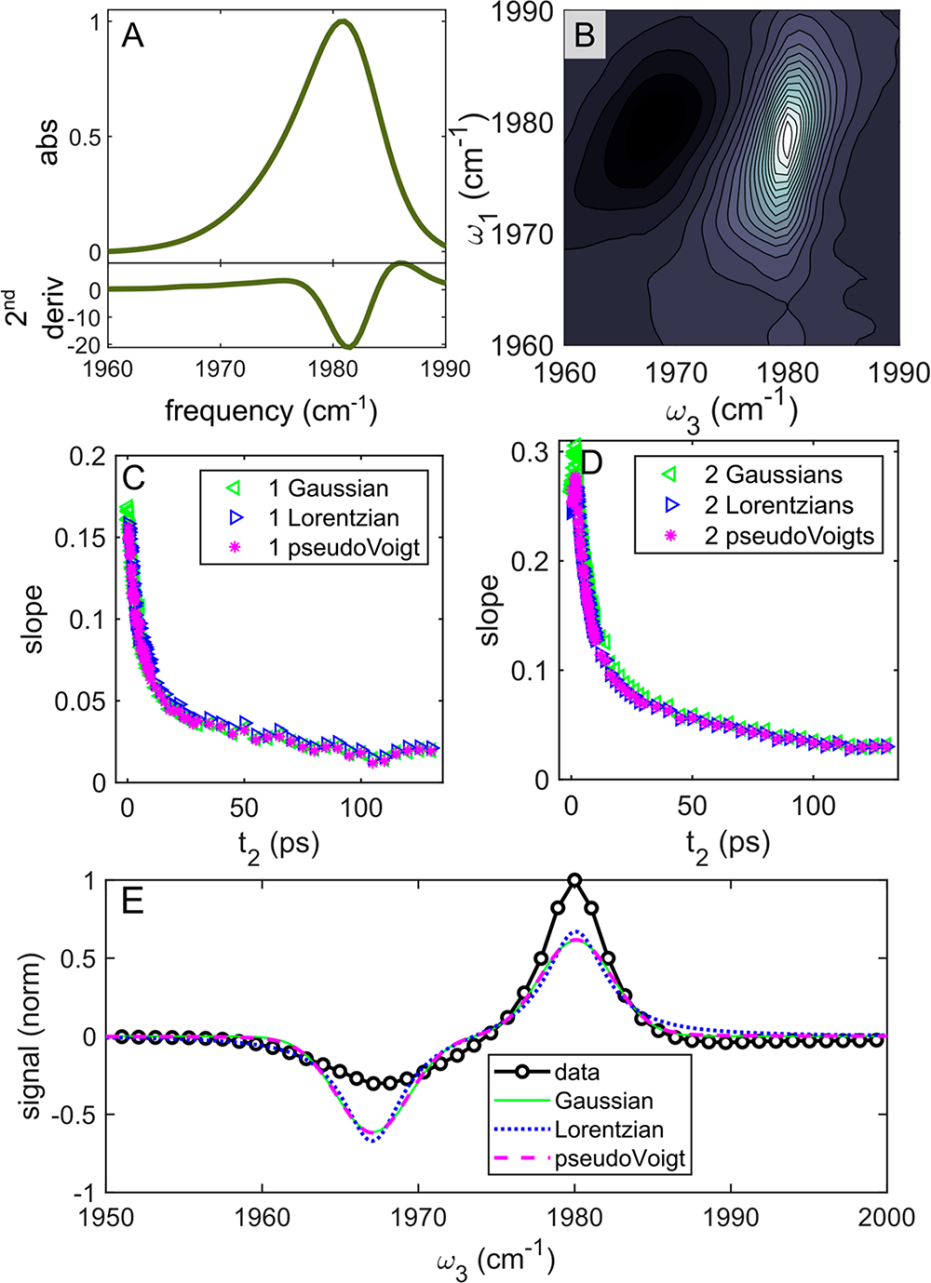
Experimental application of CLS methods. (A) Normalized FTIR spectrum of W(CO)_6_ in 1-octanol showing the absorbance (top) and the second derivative of the absorbance (bottom). (B) 2D IR spectrum from the FFCF experiment at *t*_2_ = 0.125 ps. (C) FFCFs measured from the 2D IR spectra using single peak fits. (D) FFCFs measured from the 2D IR spectra using peak pair fits. (E) Pump slice from one of the 2D spectra of W(CO)_6_ overlaid with peak pair fits, which do not reproduce the overall shape.

The experimental 2D IR and FTIR spectra for W(CO)_6_ in 1-octanol are shown in Figure 4 and Figure S5. As shown in Figure 4A,B, the line shape for the carbonyl stretch of W(CO)_6_ in 1-octanol is highly asymmetric. The integrated 2D IR signal (Supporting Information) and second derivative analysis (Figure 4A, bottom) indicate that the band is likely not the sum of two separate peaks. This asymmetry would not be captured using a standard Kubo fit, making the CLS method ideal for analyzing spectral diffusion in this system.

The results of a CLS experiment using W(CO)_6_ in 1-octanol are shown in Figure 4C,D and Table 2. The SNR was *t*_2_-dependent and varied from 180 to 38, as shown in the Supporting Information. The direct maximum and full center of mass results did not yield useful CLS data and could not be fit well to a biexponential decay. While the other methods revealed some slow component to the FFCF, there was no slow component to the direct maximum or full center of mass. In addition to noise sensitivity, the direct maximum suffers from a discretization artifact, which is particularly severe in the case of narrow peaks. Discretization effects on the direct maximum method are apparent when CLS data are overlaid with experimental spectra (Figure S8 in the Supporting Information). The discretization errors were also present in the CLS simulations but are particularly pronounced for W(CO)_6_ due to the narrow width of the CO band. The center of mass calculated from the full slice suffers primarily from the noise sensitivity observed in the simulated CLS experiments. The partial center of mass method using the data within the pump slice FWHM also failed to yield reasonable results, although we noted that it could yield a slightly more reasonable CLS decay if the cutoff was changed such that more than the FWHM is included (Figure S9 in the Supporting Information).

**Table 2:** Results from CLS Analysis of Experimental Spectra with 95% Confidence Intervals from Linear Regression Fitting

method	τ_1_ (ps)	τ_2_ (ps)	*R*^2^
1 Gaussian	5.7 ± 0.5	110 ± 30	0.9957
1 Lorentzian	4.8 ± 0.7	90 ± 20	0.9885
1 pseudoVoigt	5.4 ± 0.7	100 ± 20	0.9924

Applying peak pair fits to this system was not straightforward; due to the asymmetry of the peak and the differences between the (01) and (12) lobes of the signal, we were not able to use a Kubo fit to extract the anharmonicity, so the anharmonicity was estimated and held constant to avoid overfitting. The anharmonicity estimate used was the distance between the maximum and minimum of a representative pump slice, shown in Figure 4E. Additionally, the pair fits rely on an assumption that the (01) and (12) lobes are close to identical, and that assumption did not hold for W(CO)_6_ in octanol due to deviation from harmonic potential, as shown in Figure 4B,E and the Supporting Information. The CLS decays obtained using the peak pair methods suffered a nonphysical increase in slope values throughout the early *t*_2_ range with a maximum at 2 ps, as shown in Figure 4D. Further investigation, shown in the Supporting Information, revealed that the CLS curves corresponding to the (12) lobe contained two exponential decay components and an oscillating component, while the CLS curve corresponding to the (01) lobe only exhibited the biexponential decay. While an oscillatory component is often observed in metal carbonyl CLS data,^10,24^ the fact that it was more pronounced in one lobe than the other created an artifact through the early *t*_2_ range in the peak pair CLS curves. However, the CLS curves obtained from the peak pair methods were less noisy than the CLS curves obtained from single peaks, which is apparent when comparing the slopes as a function of *t*_2_ (Figure 4C,D) or when fitting the CLS data at later *t*_2_ (see Table S3 in the Supporting Information) to exclude the artifact from the oscillating component. Knowing the limitation of each method, the choice between single peak methods and peak pair methods may require comparing the (01) and (12) lobes as well as evaluating noise.

All single peak fits indicate a fast initial decay with a correlation lifetime of about 5–6 ps and a slower decay with a time constant near 100 ps, as shown in Table 2. Furthermore, the peak pair methods and the single peak fits to the (12) lobes also agree with the near 100 ps long decay of frequency correlations. These results are in line with what would be expected from the spectral diffusion of W(CO)_6_ in polar solvents^24,45−49^ as well as from systematic studies of spectral diffusion performed with other metal carbonyls in a series of aliphatic alcohols.^50^ As was the case for the simulated data, it does not appear to matter if the peaks are fit to Gaussians, Lorentzians, or pseudoVoigts.

## Conclusion

We have compared several methods for extracting CLS data from 2D spectra. The observed advantages and disadvantages of specific methods for determining maxima are summarized below. Peak center of mass methods can be used with high levels of noise, but they are inconsistent and must exclude the baseline to be useful. Fits with peak pairs perform the best at reducing the effects of noise but require significant assumptions about the (01) and (12) lobes of the 2D IR signal. Single peak fits using only partial pump slices tolerate noise slightly worse than peak pairs but require fewer assumptions, making them potentially more versatile in experiments.

## Supporting Information Available

Table of parameters, box plots of simulation results, supporting spectra, additional CLS curves, and supporting references (PDF)
