# Supplementary material for: An Evaluation of Maximum Determination Methods for Center Line Slope Analysis
Source: J Phys Chem B. 2023 May 9;127(19):4268–76. doi: 10.1021/acs.jpcb.2c07565 (PMC10201534; doi:10.1021/acs.jpcb.2c07565)
Supplement: Supplementary file 1 — jp2c07565_si_001.pdf [file jp2c07565_si_001.pdf]

An Evaluation of Maximum Determination Methods for Center Line Slope Analysis

Valentine, Mason L; Weisehan, Garret D; Xiong, Wei**

University of California San Diego Department of Chemistry and Biochemistry, San Diego, CA, 92093

*masonleevalentine@gmail.com

*w2xiong@ucsd.edu

Simulation Parameters 2

Supplementary Simulation Figures 5

Supplementary Experimental Figures 9

CLS of the (12) Lobe of $W(CO)_6$ in Octanol 14

CLS of $W(CO)_6$ in Octanol at With the Early t2 Artifact Excluded 16

Supplementary References 17

Simulation Parameters

Table S1: parameters for all systems used to test noise effects on CLS methods

system number	Δanh (cm^{-1})	τ_1 (ps)	τ_2 (ps)	ω (cm^{-1})	$\Delta\omega_1$ (cm^{-1})	$\Delta\omega_2$ (cm^{-1})	Lifetime (ps)	dephasing time (ps)
1	27.01	2.103	38.62	2045.9	5.403	6.812	10.063	2.881
2	23.44	2.939	20.82	2040.1	3.468	5.292	49.359	2.484
3	40.88	1.820	26.14	2058.7	6.779	9.768	66.593	0.879
4	20.84	2.798	37.59	2040.2	9.530	5.182	39.012	2.408
5	22.17	2.659	20.05	2055.2	8.226	5.521	11.757	0.709
6	43.12	1.457	35.48	2057.8	7.631	3.297	33.441	2.380
7	20.20	2.702	26.58	2054.8	4.203	2.098	95.583	2.238
8	21.81	2.178	16.13	2036.0	9.892	9.697	86.184	1.581
9	36.57	2.583	22.61	2048.1	9.702	4.350	33.963	2.302
10	21.47	2.452	34.75	2070.0	8.975	8.453	50.261	1.822
11	25.17	1.926	16.77	2049.1	5.987	8.668	35.257	0.609
12	51.19	2.282	34.72	2063.6	8.314	9.913	3.810	2.390
13	22.43	1.441	20.92	2060.6	7.293	8.763	7.148	0.517
14	18.03	0.929	36.78	2035.9	8.078	5.257	63.151	0.690
15	46.87	2.768	17.60	2066.0	7.518	9.749	41.414	2.808
16	29.74	0.871	28.99	2053.2	6.079	7.091	81.745	2.479
17	33.79	1.378	29.73	2057.1	4.183	4.920	80.925	2.876
18	32.44	2.945	12.98	2062.8	8.990	8.229	90.670	1.381
19	54.12	2.126	39.99	2064.7	4.656	3.674	46.775	1.978
20	15.47	0.975	31.66	2041.8	9.371	7.610	10.957	1.143
21	24.71	1.484	20.57	2035.5	3.647	2.368	57.742	2.622
22	29.31	0.791	22.60	2039.7	3.611	4.124	63.610	1.252
23	32.14	2.101	16.80	2058.7	3.398	9.217	6.967	2.502
24	15.44	0.911	20.61	2037.4	2.429	9.440	60.632	2.877
25	39.40	2.926	14.86	2042.7	3.561	9.995	21.830	1.181
26	57.68	0.785	36.61	2045.3	6.801	4.024	60.451	1.008
27	45.77	1.091	19.91	2032.5	7.834	6.783	5.239	2.482
28	50.89	2.821	26.87	2053.0	3.732	8.016	68.605	1.803
29	43.75	2.881	10.78	2040.3	4.750	9.198	85.922	1.949
30	16.49	1.364	32.62	2063.9	7.167	8.014	68.936	0.550
31	25.67	1.489	20.53	2037.6	5.380	4.477	63.883	0.928
32	19.73	1.432	19.48	2043.3	9.737	5.437	73.141	0.979
33	25.62	2.070	29.17	2065.6	7.239	5.043	31.099	1.242
34	54.89	1.088	37.15	2031.9	8.717	9.355	94.137	1.256
35	31.33	1.443	21.87	2064.2	8.158	5.931	99.917	1.519
36	48.07	2.767	24.88	2063.6	7.175	5.589	29.996	0.724
37	47.38	2.746	29.26	2040.2	6.397	4.616	17.019	2.779
38	52.64	2.264	23.81	2040.3	3.222	7.167	19.912	2.448
39	25.14	1.782	17.81	2042.0	5.245	9.457	20.936	2.421

40	43.32	1.308	21.63	2045.9	8.967	2.847	66.447	1.711
41	19.83	2.237	12.06	2069.5	2.477	5.736	5.299	2.917
42	34.54	0.989	27.42	2040.9	5.784	7.730	55.391	1.008
43	37.13	2.245	13.51	2057.7	5.628	9.460	77.521	0.526
44	56.69	0.982	22.27	2067.9	7.370	5.703	16.308	0.694
45	35.58	1.650	36.83	2050.0	3.836	3.335	32.433	2.009
46	36.23	2.496	34.63	2034.8	8.058	2.176	46.723	1.121
47	47.50	2.665	11.90	2031.4	5.651	2.971	45.517	2.756
48	15.75	2.801	19.26	2039.7	4.769	5.567	13.507	1.808
49	52.99	2.261	37.85	2046.3	2.878	3.097	12.206	2.490
50	56.49	1.740	20.60	2052.7	3.494	5.248	37.753	0.747
51	56.81	2.444	12.71	2049.2	5.950	9.366	17.304	2.597
52	49.50	1.961	14.32	2039.2	6.824	7.541	4.197	1.308
53	41.81	2.002	17.79	2065.4	5.686	2.385	63.021	2.839
54	43.46	2.356	11.06	2068.7	5.347	2.370	62.334	1.658
55	25.19	2.898	28.15	2053.8	8.049	2.986	98.814	0.671
56	23.95	1.264	19.52	2068.2	4.657	7.317	80.806	0.694
57	18.55	0.880	21.13	2045.3	6.086	4.197	39.993	2.486
58	19.51	1.098	11.59	2033.9	8.539	7.027	49.502	1.657
59	39.24	1.570	30.49	2053.1	4.351	7.200	89.524	2.476
60	15.51	2.179	11.84	2031.3	8.234	7.565	1.022	1.719
61	26.04	2.101	29.11	2055.9	6.676	2.059	11.332	2.611
62	21.78	1.006	25.59	2058.5	9.707	5.256	53.455	2.269
63	54.95	2.995	22.80	2045.1	7.716	6.971	30.681	2.210
64	26.69	0.848	16.62	2043.5	5.697	4.559	37.380	1.538
65	58.31	2.086	23.45	2046.0	4.903	5.319	54.655	2.336
66	59.06	2.416	39.30	2060.8	9.509	8.648	24.321	1.876
67	42.93	2.914	38.68	2053.0	6.037	2.325	22.892	1.226
68	16.75	2.189	20.65	2068.5	6.520	7.117	1.709	1.742
69	29.33	2.261	13.72	2047.2	7.455	9.846	34.385	1.458
70	48.60	2.367	39.52	2066.2	2.932	2.617	84.421	1.307
71	35.98	2.354	23.58	2030.1	2.536	2.366	50.621	1.110
72	31.31	2.686	37.73	2067.3	4.210	2.753	81.858	2.093
73	40.71	0.752	18.96	2039.1	7.853	3.314	57.000	2.138
74	16.84	1.123	14.42	2049.1	5.904	6.134	36.146	0.992
75	29.42	2.516	11.84	2060.9	3.902	7.559	33.438	2.818
76	37.83	1.303	13.06	2050.9	5.549	7.588	95.846	2.047
77	16.83	2.503	27.92	2049.1	8.022	4.766	90.612	2.134
78	38.68	2.061	27.83	2041.2	8.806	3.507	32.356	2.441
79	38.89	1.557	37.15	2039.8	8.883	4.691	91.956	1.426
80	56.42	1.583	16.29	2042.5	3.926	3.166	38.189	2.069
81	49.76	1.832	11.56	2047.2	3.652	7.678	53.967	2.442
82	21.50	1.348	25.52	2034.3	2.944	9.575	33.404	0.594
83	32.92	2.965	17.39	2040.7	6.527	8.073	17.698	2.861
	34.57	0.868	22.08	2031.9	4.469	5.536	42.438	1.637

84								
85	23.60	0.843	28.85	2058.3	3.827	2.592	40.442	1.289
86	26.35	1.046	26.53	2056.4	5.268	5.921	11.108	0.737
87	25.91	2.341	34.25	2034.2	9.998	8.653	85.945	1.091
88	59.68	1.618	29.63	2052.5	6.094	4.102	82.820	1.623
89	32.48	2.523	20.75	2052.4	2.790	6.506	14.209	2.372
90	42.69	2.421	14.96	2040.6	8.967	6.964	8.021	2.607
91	47.91	2.045	10.37	2061.1	4.681	7.910	99.218	0.987
92	23.38	1.764	10.88	2052.9	5.262	7.336	43.455	2.861
93	36.91	2.423	14.83	2060.0	9.705	2.495	83.276	2.024
94	31.86	2.613	18.51	2060.1	3.995	5.140	57.162	2.933
95	56.72	2.543	16.09	2057.4	8.899	6.032	48.503	0.764
96	52.55	2.273	12.26	2036.8	4.398	9.032	78.769	1.729
97	18.26	1.818	28.36	2031.9	4.422	2.065	82.926	2.800
98	57.61	1.459	33.57	2035.4	9.216	8.561	24.570	0.866
99	50.90	2.786	30.22	2052.8	3.724	3.979	4.857	1.416
100	44.60	2.399	12.43	2066.1	9.141	4.167	61.697	2.540

Supplementary Simulation Figures

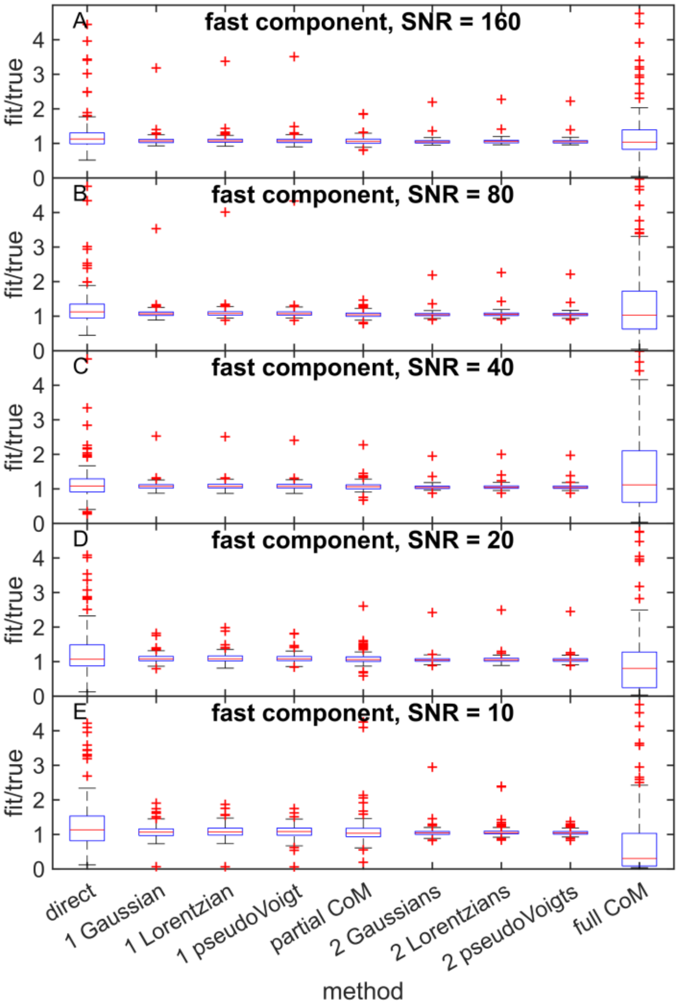

Figure S1: Noise test results for the fast component. (A) box plot of fit/true values obtained with all methods for SNR = 160. (B) box plot of fit/true values obtained with all methods for SNR = 80. (C) box plot of fit/true values obtained with all methods for SNR = 40. (D) box plot of fit/true values obtained with all methods for SNR = 20. (E) box plot of fit/true values obtained with all methods for SNR = 10. Not all outliers are shown for the direct or full center-of-mass methods.

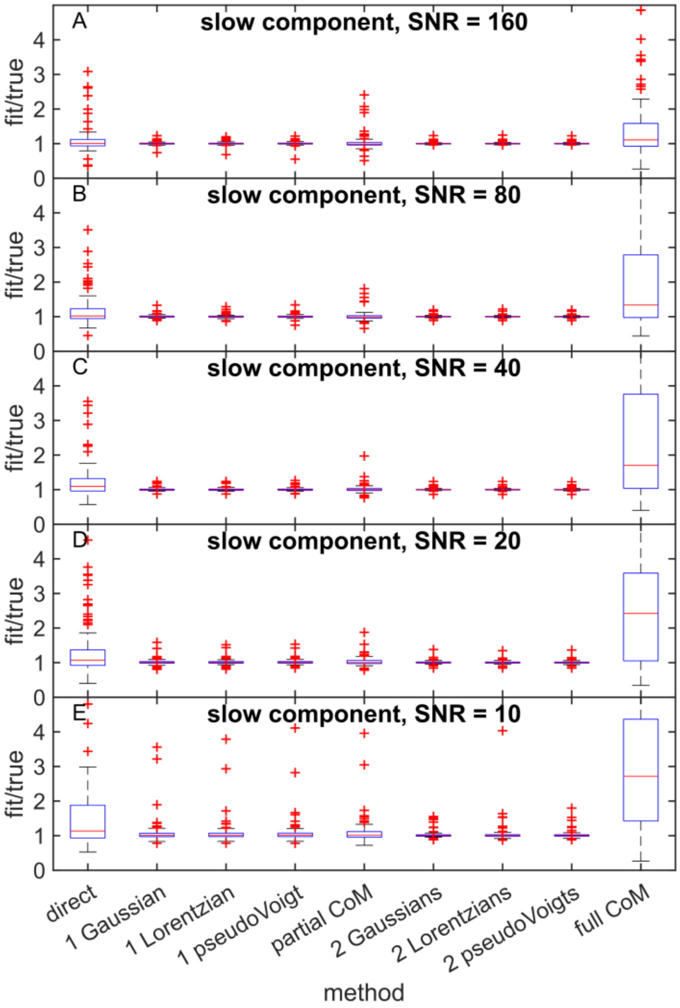

Figure S2: Noise test results for the slow component. (A) box plot of fit/true values obtained with all methods for SNR = 160. (B) box plot of fit/true values obtained with all methods for SNR = 80. (C) box plot of fit/true values obtained with all methods for SNR = 40. (D) box plot of fit/true values obtained with all methods for SNR = 20. (E) box plot of fit/true values obtained with all methods for SNR = 10. Not all outliers are shown for the direct or full center-of-mass methods.

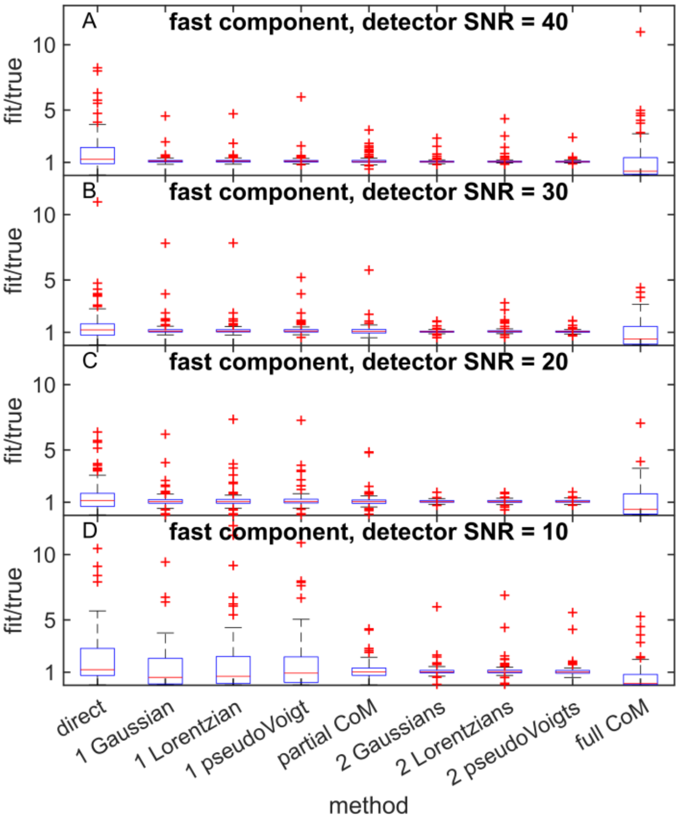

Figure S3: Detector noise test results for the fast component. (A) box plot of fit/true values obtained with all methods for detector SNR = 40. (B) box plot of fit/true values obtained with all methods for detector SNR = 30. (C) box plot of fit/true values obtained with all methods for detector SNR = 20. (D) box plot of fit/true values obtained with all methods for detector SNR = 10. Not all outliers are shown for the direct or full center-of-mass methods.

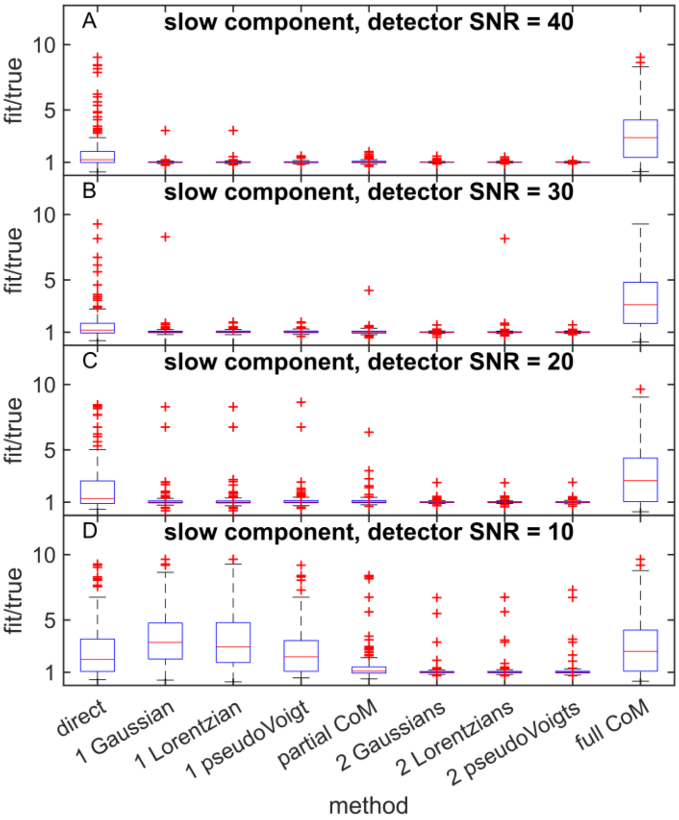

Figure S4: Detector noise test results for the slow component. (A) box plot of fit/true values obtained with all methods for detector SNR = 40. (B) box plot of fit/true values obtained with all methods for detector SNR = 30. (C) box plot of fit/true values obtained with all methods for detector SNR = 20. (D) box plot of fit/true values obtained with all methods for detector SNR = 10. Not all outliers are shown for the direct or full center-of-mass methods.

Supplementary Experimental Figures

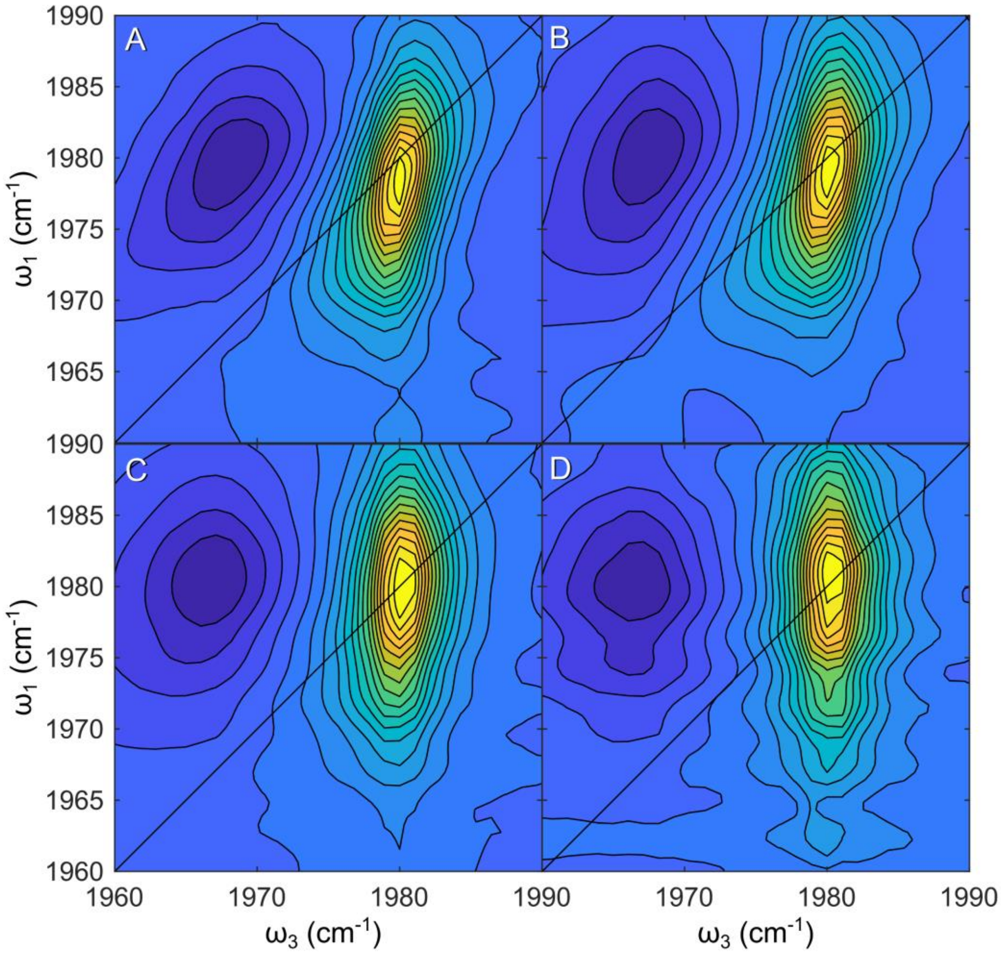

Figure S5: time-dependent 2D IR spectra of $W(CO)_6$ in 1-octanol. Representative 2D IR spectra of the carbonyl stretching vibration at t_2 = 0.125 ps (A), t_2 = 1.00 ps (B), t_2 = 10.00 ps (C), and t_2 = 100.00 ps (D).

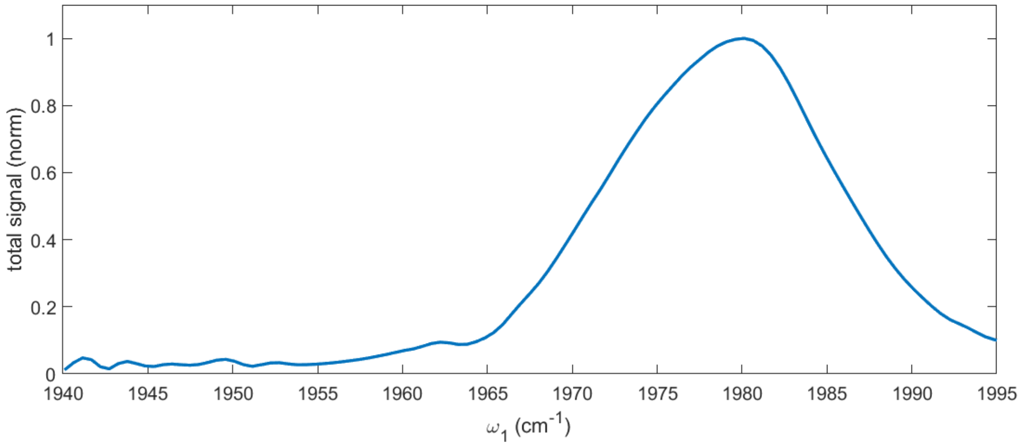

Figure S6: Pump-slice amplitude spectrum of $W(CO)_6$ in octanol. The spectrum was calculated by integrating the absolute value of the 2D IR signal at each pump frequency. The 2D spectrum is asymmetric, but with no clear separation between peaks.

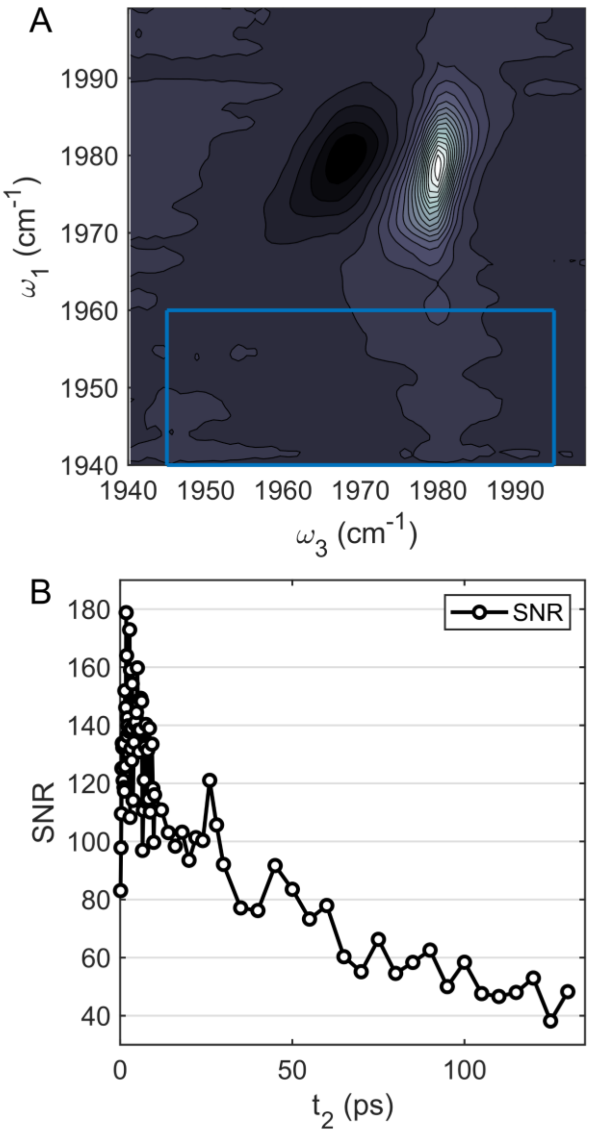

Figure S7: Estimation of noise in experimental 2D spectra. (A) 2D IR spectrum of $W(CO)_6$ at t2 = 0.125 ps with the region used for noise estimation highlighted. The Region extends from 1940 to 1960 cm^{-1} in ω_1 and from 1945 to 1995 cm^{-1} in ω_3. (B) Results from SNR estimation. SNR was estimated as the ratio between the maximum signal and the standard deviation of the noise estimation region.

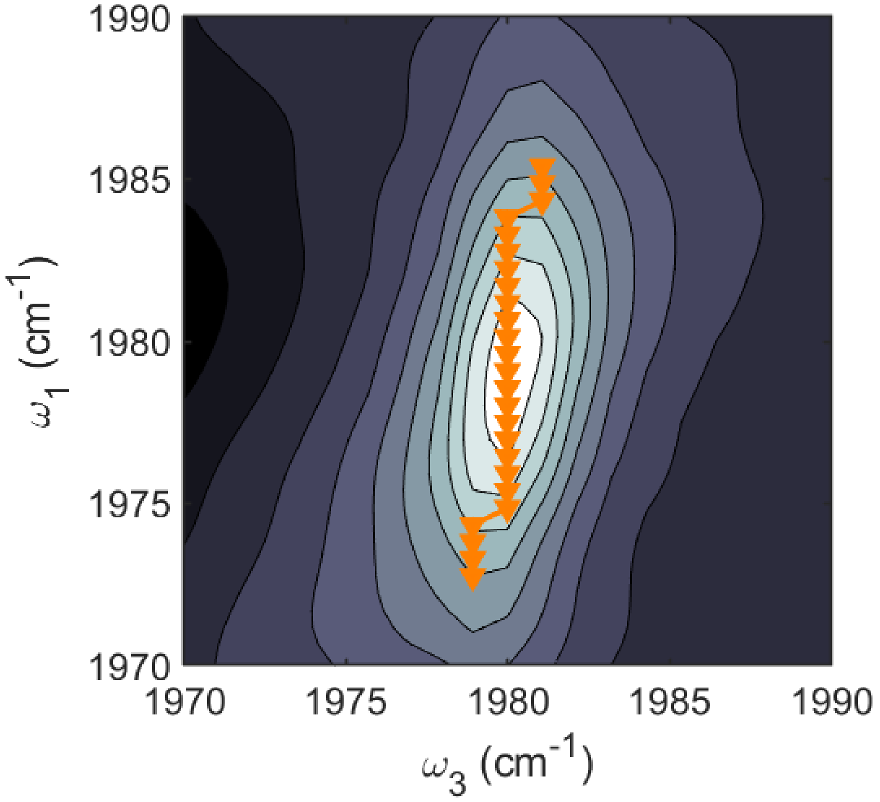

Figure S8: direct maximum CLS data overlaid with an experimental 2D spectrum showing pixelated and discrete maxima.

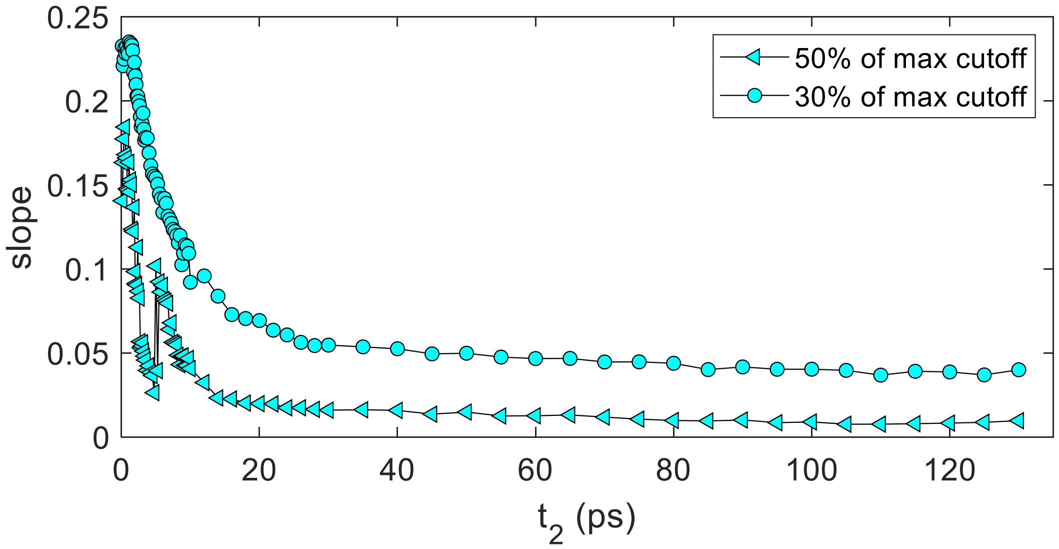

Figure S9: Partial center-of-mass CLS of $W(CO)_6$ in octanol. The CLS curve could be made to look more reasonable by changing the cutoff from 50% of the maximum to some other value, however, the CLS decay with the center-of-mass does not yeild a precise correlation time for the slow component.

CLS of the (12) Lobe of $W(CO)_6$ in Octanol

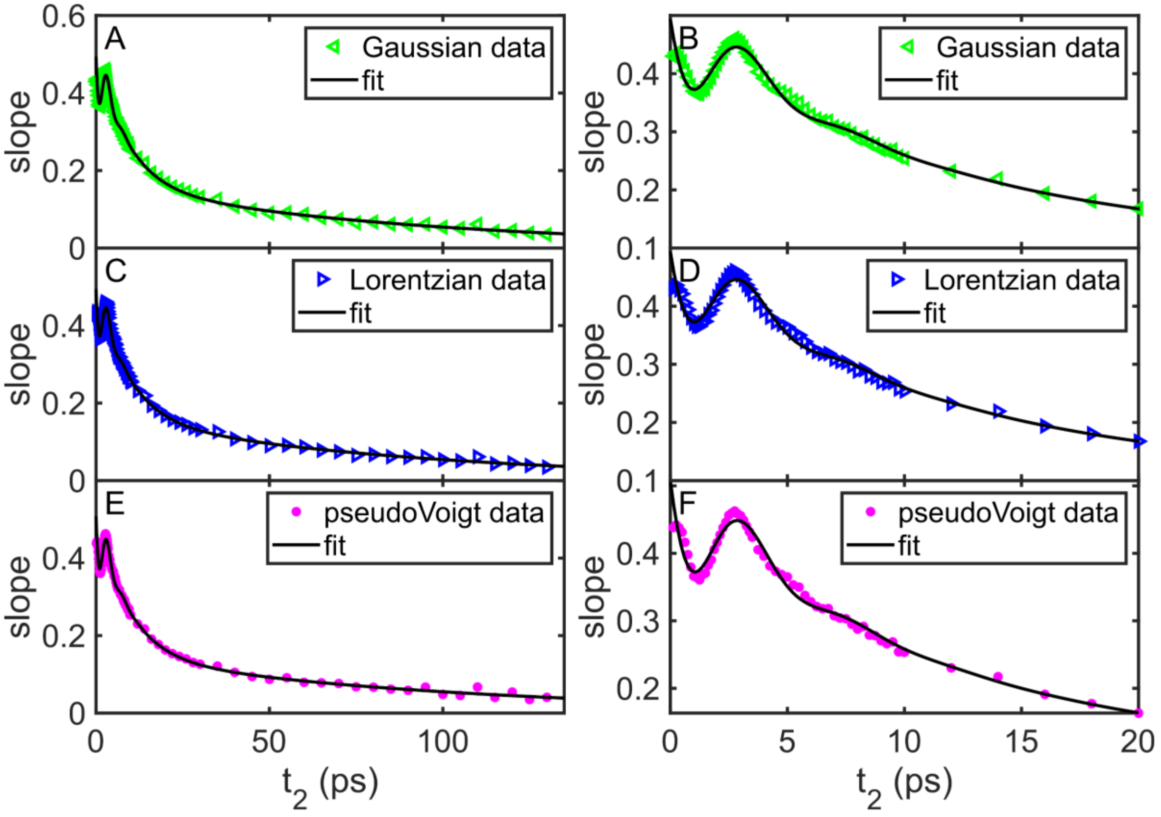

Figure S10: CLS of the (12) lobe of $W(CO)_6$ in octanol fit to the sum of two exponential terms and a damped oscillator term. The full t2 range is shown on the left and a detail of the early decay is shown on the right. (A-B) Slopes obtained with Gaussian peak fits and the fit to the slopes. (C-D) Slopes obtained with Lorentzian peak fits and the fit to the slopes. (E-F) Slopes obtained with pseudoVoigt peak fits and the fit to the slopes.

The (12) lobe of the 2D IR signal was analyzed using the single-peak methods. Due to oscillations in the data, the data was fit to the sum of a biexponential decay and a damped oscillator term.

$$C(t_2) = A_1 e^{-t_2/\tau_1} + A_2 e^{-t_2/\tau_2} + A_3 e^{-t_2/\tau_3} \cos\left(\frac{2\pi}{T_{osc}} t_2 + \phi\right)$$

Eq S1

Where τ_1, τ_2, and τ_3 are decay lifetimes in picoseconds, A_1, A_2, and A_3 are scaling constants, T_{osc} is the period of the oscillatory term in ps, and φ is a phase shift constant. Similar oscillatory terms have been included in CLS data for a variety of systems including metal carbonyls in aliphatic alcohols.[1–5]

Table S2: Fitting Results from the CLS of the (12) lobes of $W(CO)_6$ with 95% confidence intervals from linear regression fitting

Method	τ_1 (ps)	τ_2 (ps)	τ_3 (ps)	T_{osc} (ps)	R^2
1 Gaussian	8 ± 1	90 ± 15	1.9 ± 0.5	4.9 ± 0.3	0.9965
1 Lorentzian	9 ± 1	90 ± 15	2.0 ± 0.4	4.9 ± 0.3	0.9964
1 pseudoVoigt	9 ± 1	100 ± 20	2.1 ± 0.5	4.8 ± 0.3	0.9951

CLS of $W(CO)_6$ in Octanol at With the Early t_2 Artifact Excluded

Table S3: Fitting Results from the CLS of $W(CO)_6$ in octanol only using data with t_2 greater than 5 ps with 95% confidence intervals from linear regression fitting.

Method	τ_1 (ps)	τ_2 (ps)	R^2
1 Gaussian	6 ± 2	120 ± 30	0.9861
1 Lorentzian	8 ± 2	120 ± 30	0.9912
1 pseudoVoigt	7 ± 2	110 ± 30	0.9886
2 Gaussians	7.1 ± 0.8	100 ± 10	0.9984
2 Lorentzians	6.4 ± 0.7	100 ± 10	0.9982
2 pseudoVoigts	6.3 0.7	100 ± 10	0.9981

Supplementary References

(1) Pagano, P.; Guo, Q.; Ranasinghe, C.; Schroeder, E.; Robben, K.; Häse, F.; Ye, H.; Wickersham, K.; Aspuru-Guzik, A.; Major, D. T.; Gakhar, L.; Kohen, A.; Cheatum, C. M. Oscillatory Active-Site Motions Correlate with Kinetic Isotope Effects in Formate Dehydrogenase. *ACS Catal.* **2019**, *9* (12), 11199–11206. https://doi.org/10.1021/acscatal.9b03345.

(2) Wong, D. B.; Giammanco, C. H.; Fenn, E. E.; Fayer, M. D. Dynamics of Isolated Water Molecules in a Sea of Ions in a Room Temperature Ionic Liquid. *J. Phys. Chem. B* **2013**, *117* (2), 623–635. https://doi.org/10.1021/jp310086s.

(3) Schmidt-Engler, J. M.; von Berg, S.; Bredenbeck, J. Temperature-Dependent Low-Frequency Modes in the Active Site of Bovine Carbonic Anhydrase II Probed by 2D-IR Spectroscopy. *J. Phys. Chem. Lett.* **2021**, *12* (32), 7777–7782. https://doi.org/10.1021/acs.jpclett.1c01453.

(4) Nishida, J.; Tamimi, A.; Fei, H.; Pullen, S.; Ott, S.; Cohen, S. M.; Fayer, M. D. Structural Dynamics inside a Functionalized Metal–Organic Framework Probed by Ultrafast 2D IR Spectroscopy. *Proc. Natl. Acad. Sci.* **2014**, *111* (52), 18442–18447. https://doi.org/10.1073/pnas.1422194112.

(5) Duan, R.; Mastron, J. N.; Song, Y.; Kubarych, K. J. Direct Comparison of Amplitude and Geometric Measures of Spectral Inhomogeneity Using Phase-Cycled 2D-IR Spectroscopy. *J. Chem. Phys.* **2021**, *154* (17), 174202. https://doi.org/10.1063/5.0043961.
